# A1M Ameliorates Preeclampsia-Like Symptoms in Placenta and Kidney Induced by Cell-Free Fetal Hemoglobin in Rabbit

**DOI:** 10.1371/journal.pone.0125499

**Published:** 2015-05-08

**Authors:** Åsa Nääv, Lena Erlandsson, Josefin Axelsson, Irene Larsson, Martin Johansson, Lena Wester-Rosenlöf, Matthias Mörgelin, Vera Casslén, Magnus Gram, Bo Åkerström, Stefan R. Hansson

**Affiliations:** 1 Obstetrics and Gynecology, Department of Clinical Sciences Lund, Lund University, Lund, Sweden; 2 Nephrology, Department of Clinical Sciences Lund, Lund University, Lund, Sweden; 3 Clinical Pathology, Department of Laboratory Medicine, Lund University, Malmö, Sweden; 4 Infection Medicine, Department of Clinical Sciences Lund, Lund University, Lund, Sweden; Centre Hospitalier Universitaire Vaudois, FRANCE

## Abstract

Preeclampsia is one of the most serious pregnancy-related diseases and clinically manifests as hypertension and proteinuria after 20 gestational weeks. The worldwide prevalence is 3-8% of pregnancies, making it the most common cause of maternal and fetal morbidity and mortality. Preeclampsia lacks an effective therapy, and the only “cure” is delivery. We have previously shown that increased synthesis and accumulation of cell-free fetal hemoglobin (HbF) in the placenta is important in the pathophysiology of preeclampsia. Extracellular hemoglobin (Hb) and its metabolites induce oxidative stress, which may lead to acute renal failure and vascular dysfunction seen in preeclampsia. The human endogenous protein, α_1_-microglobulin (A1M), removes cell-free heme-groups and induces natural tissue repair mechanisms. Exogenously administered A1M has been shown to alleviate the effects of Hb-induced oxidative stress in rat kidneys. Here we attempted to establish an animal model mimicking the human symptoms at stage two of preeclampsia by administering species-specific cell-free HbF starting mid-gestation until term, and evaluated the therapeutic effect of A1M on the induced symptoms. Female pregnant rabbits received HbF infusions i.v. with or without A1M every second day from gestational day 20. The HbF-infused animals developed proteinuria and a significantly increased glomerular sieving coefficient in kidney that was ameliorated by co-administration of A1M. Transmission electron microscopy analysis of kidney and placenta showed both intracellular and extracellular tissue damages after HbF-treatment, while A1M co-administration resulted in a significant reduction of the structural and cellular changes. Neither of the HbF-treated animals displayed any changes in blood pressure during pregnancy. In conclusion, infusion of cell-free HbF in the pregnant rabbits induced tissue damage and organ failure similar to those seen in preeclampsia, and was restored by co-administration of A1M. This study provides preclinical evidence supporting further examination of A1M as a potential new therapy for preeclampsia.

## Introduction

Preeclampsia is a pregnancy specific clinical syndrome that manifests during the second half of pregnancy and is one of the leading causes of maternal mortality and morbidity [1, 2]. The disease is characterized by *de novo* hypertension with proteinuria manifesting after 20 gestational weeks [3]. It is also associated with general endothelial damage and glomerular endotheliosis, characterized by occlusion of capillary lumen, glomerular endothelial swelling and loss of endothelial fenestration [4–6]. This leads to disruption of the filtration barrier in the kidneys with subsequent proteinuria [7]. Preeclampsia is probably the most common glomerular disease in the world afflicting approximately 3–8% of all pregnancies [6]. To date the only treatment available is symptomatic blood pressure treatment and the only known cure is delivery. Hence, preeclampsia is an important cause in ~15% of pre-term deliveries. Also, 25% of preeclampsia cases lead to intrauterine growth retardation (IUGR) of the fetus. Both of these conditions result in infant morbidity and substantial health care expenditure [8].

The etiology of preeclampsia remains unknown but the disease is believed to evolve in two stages [9]. The first stage is characterized by a defective placentation through incomplete conversion of the spiral arteries [10]. This results in uneven blood perfusion and oxidative stress. Stage two of the disease is characterized by clinical manifestations and symptoms based on maternal endothelial damage and systemic inflammation that are suggested to be caused by placental-derived material such as trophoblast debris, micro vesicles and micro-RNA [5, 11–13].

It has been shown that cell-free fetal hemoglobin (HbF) is an important factor in the pathogenesis of preeclampsia [14, 15]. With the use of microarray and proteomic technologies, healthy and preeclamptic placentas were compared. An increased expression of HbF, seen both as elevated mRNA levels in hematopoietic stem cells and as accumulation of the protein in vascular lumen, was seen in preeclampsia [14]. Cell-free hemoglobins (Hb), HbF as well as adult Hb (HbA), are strongly redox active molecules causing oxidative stress by formation of free heme, iron and generation of free radicals [16–18]. This potentially leads to damage to the placental barrier, allowing fetal factors including HbF to leak into the maternal bloodstream, thereby causing endothelial disruption and vasoconstriction. This hypothesis was supported *ex-vivo* using the human placenta perfusion model [15]. Cell-free HbA was added to the fetal circulation, resulting in increased circulatory pressure and placental barrier damage with subsequent leakage from the fetal circulation into the maternal circulation. Transmission electron microscopy revealed placental tissue damage typical to those observed in preeclamptic placentas [15, 19]. These included damage to the extra cellular matrix with an almost complete loss of collagen fibrils, cellular changes to membranes, nuclei and mitochondria. Gene profiling of Hb-perfused placentas displayed a similar genetic profile as placentas from women with preeclampsia. These *ex vivo* findings suggest that free Hb plays an important role in the disease etiology [15, 20]. To study the systemic effect of placental derived cell-free HbF *in vivo* with regards to kidney function, one of the hallmarks of preeclampsia, cell-free HbF was infused into rats. The glomerular permeability to macromolecules was increased as a result [21].

Several human endogenous defense mechanisms exist to protect against the harmful effects of extracellular Hb, among them is the plasma and tissue protein α_1_-microglobulin (A1M) that contributes to minimize adverse effects of cell-free Hb *in vivo*. The A1M protein is primarily produced in the liver, distributed to the extracellular fluids and compartments via the blood circulation, and is present mainly in extracellular fluids. It has properties as an antioxidant as well as a binding protein for free radicals and heme [22–24]. Elevated levels of cell-free Hb, free radicals, and heme up-regulate the synthesis of A1M [25]. Increased A1M levels have been observed in the preeclamptic placenta, suggested to be up-regulated in defense against oxidative stress [18]. A1M prevents oxidation and oxidative damage to cells and matrix molecules, and removes heme from cell membranes and cytosol [23, 26].

The protective effects and the therapeutic potential of A1M in preeclampsia have been studied *ex vivo* in the placental perfusion model as well as in two different *in vivo* animal models. In the placenta perfusion model the toxic effects of the cell-free Hb administered to the fetal circulation was ameliorated by co-administration of A1M to the maternal circulation [15]. In a rat *in vivo* model the increased glomerular permeability in the kidneys induced by infusion of cell-free HbF was restored to normal levels by A1M treatment [21]. In a second animal model preeclampsia-like symptoms were induced by starvation in ewes, and associated with elevated levels of cell-free heme [27]. Intravenous (i.v.) infusion with A1M ameliorated the structural tissue damages seen in both kidney and placenta, as well as restored the glomerular filtration rate in the kidney [28].

The purpose of the present study was to 1) further elucidate the role of cell-free HbF in the pathogenesis of preeclampsia and 2) evaluate the therapeutic potential of A1M to relieve preeclampsia symptoms. We attempted to establish a rabbit model mimicking the human symptoms at stage two of preeclampsia by administering species-specific cell-free HbF starting mid-gestation until term, and evaluated the therapeutic effect of A1M on the induced symptoms.

## Materials and Methods

### Pregnant rabbit model

**Fig 1:**
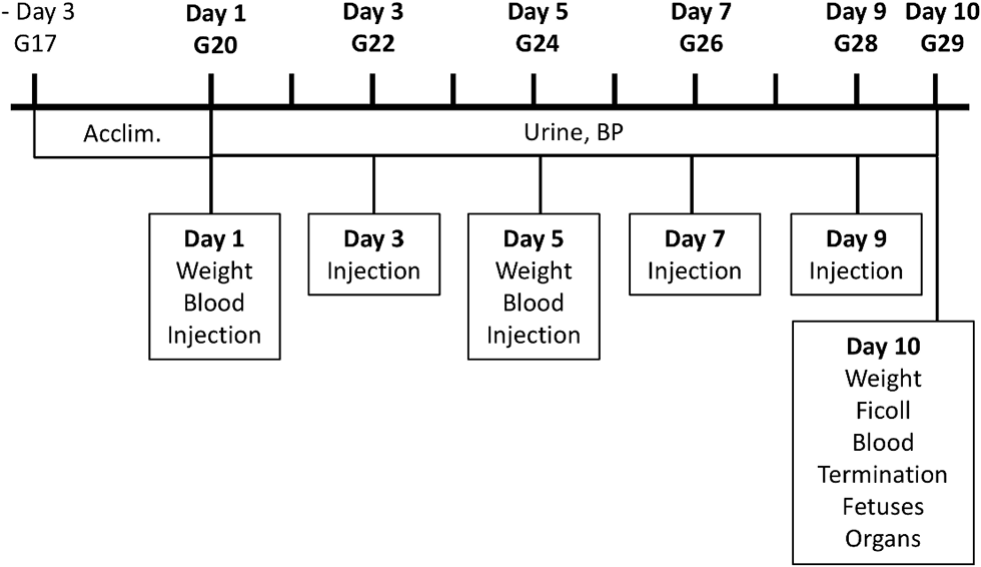
Experimental design. Illustrated is the experimental design with time points for collection of urine and blood, blood pressure measurements, injections and weight measurements during the 10 days experimental period.

The study was approved by the ethical committee for animal studies at Lund University, Sweden (permission no: M58-12). The experimental design is illustrated in Fig 1. Pregnant rabbits (New Zealand White/Loop cross) arrived to the animal facility at gestational day 17 (G17) and were randomly assigned to the study groups. All rabbits were acclimatized to handling procedures for 3 days prior to the experiment. From arrival until time of termination they were fed *ad libitum* with water, pelleted food, hay, carrots and cucumbers. The experiment lasted for 10 days, from G20 until termination at G29. In an initial pilot study, two doses of cell-free HbF were tested: 10 mg/kg body weight (n = 4) and 40 mg/kg (n = 4). The low dose did not induce any measurable symptoms and the high dose was not well tolerated by the dams. For this reason, 20 mg/kg was chosen for HbF injections during the experiment. A1M doses were extrapolated from previous studies in sheep, rats and mice. A total of 19 pregnant rabbits were used for the experiment. The control rabbits received buffer only (n = 5), rabbits received HbF at 20 mg/kg (HbF-group, n = 8) and rabbits received both 20 mg/kg of HbF and 6 mg/kg of A1M (HbF/A1M-group, n = 6). Urine was collected non-invasively every morning throughout the experiment, followed by non-invasive blood pressure measurements. All rabbits were weighed and blood samples were collected at G20, G24 and G29. Injections were given i.v. in the lateral ear vein every second day (G20, G22, G24, G26 and G28). When blood sampling and injections occurred the same day, blood was collected before the injections were administered. At time of termination, animals were anesthetized, kidney function evaluated and the animals sacrificed and fetuses and organs collected.

### HbF

Rabbit fetal blood was collected from euthanized rabbit fetuses at day 29 of gestation (G29) in tubes containing heparin and centrifuged at 2000 x g for 20 minutes. Red blood cells (RBC) were collected and washed with 1x PBS buffer. The RBC´s were lysed by re-suspension in hypotonic buffer (H_2_O:PBS, 20:1) on ice. Membranes were separated from cytosol by centrifugation at 14 000 x g for 20 minutes at 4°C. The supernatant was dialyzed overnight (Spectra-Por Dialysis membrane 4, Spectrumlabs, VWR) against 15 mM Tris-HCl pH 8.0 at 4°C, centrifuged at 14 000 x g for 20 minutes at 4 °C and filtered through a Bottle Top filter (0,22 μm PES membrane, Corning) by vacuum. The supernatant was applied to a FPLC (ÄKTA, GE Healthcare) by running it through a Hiscale 26/20 column (VWR) packed with UNOsphere Q ion exchange media (Bio-Rad) in 15 mM Tris-HCl pH 8.0 buffer. Bound HbF was eluted with a 0–35% 15 mM Tris-HCl pH 8.0, 1 M NaCl- gradient and the collected fractions evaluated by SDS-PAGE (Mini-PROTEAN TGX gels, Bio-Rad). HbF-containing fractions were pooled and endotoxins removed by using an EndoTrap kit (EndoTrap blue 10, Hyglos) followed by an evaluation of the resulting endotoxin level with a LAL test (Limulus Amebocyte Lysate QCL-1000, Lonza). The final concentration of HbF was 52 mg/ml in 15 mM Tris-HCl pH 8.0, 20 mM NaCl. The HbF solution was sterile filtered and frozen at -20 °C until use. For the control animals, a sham solution containing sterile 15 mM Tris-HCl pH 8.0, 20 mM NaCl was used.

### A1M

Recombinant human A1M was prepared and donated by A1M Pharma AB (Lund, Sweden), dissolved in 10 mM Tris-HCl pH 8.0, 0,125 M NaCl at a concentration of 5 mg/ml. The A1M solution contained the full polypeptide of plasma A1M preceded by an N-terminal His8-tag [29]. The protein was tested and found to be fully functional (reduction of the ABTS-radical [24]). The A1M solution was sterile filtered and frozen at -20 °C until use. For the control animals, a sham solution containing sterile 10 mM Tris-HCl pH 8.0, 0.125 M NaCl was used.

### Blood pressure

Throughout the ten day experiment, the systolic and diastolic blood pressures were measured every morning in a calm environment using a non-invasive method. The right front leg was shaved and a blood pressure cuff (NIBP Single use cuff, Neonatal #1, DigiCare Animal Health) was applied and connected to a blood pressure monitor (Multiparameter Physiologic Monitor, DigiCare Animal Health). Mean arterial pressure (MAP) and heart rate was also determined via the monitor.

### End point and samples collected

Pregnant rabbits were anesthetized (s.c. injection, 1 ml/kg body weight of 100 mg/ml Ketalar and 1 mg/ml Dormitor) and laparotomy was performed with the pregnant rabbit in a dorsal position as previously described [30]. The fetuses were delivered, immediately euthanized and thereafter counted, weighed and measured. Blood from the mother was collected via heart puncture. Urine was collected directly from the bladder using a syringe. The mothers were euthanized by an i.v. lethal dose of Ketalar + Dormitor and organs immediately collected (liver, kidney and placenta). The organs were dissected and biopsies were fresh-frozen on dry ice, paraffin-embedded or fixed for electron microscopy as appropriate.

### Blood and urine analysis

Blood was collected from the lateral ear vein at day 1 (G20), day 5 (G24) and via heart-puncture at day 10 (G29) in EDTA- and Li-Heparin tubes. Whole blood was collected in EDTA tubes and analyzed for blood cell counts (white blood cells (WBC), lymphocytes (LYM), monocytes (MON), neutrophils (NEU), red blood cells (RBC), platelets (PL), Hemoglobin (HGB), hematocrit (HCT), mean cell volume (MCV), mean cell hemoglobin (MCH)) using a VetScan HM5 instrument set for rabbit (Abaxis Inc.). The plasma was separated from whole blood collected in Li-Heparin tubes and analyzed for liver transaminase enzyme activity (ALAT, ASAT), lactate dehydrogenase (LDH), urate and creatinine, using a Cobas Instrument (Roche). The plasma was also analyzed for glucose levels by using Contour test strips and a Contour Blood Glucose Monitoring device (Bayer Contour). The level of haptoglobin in plasma was measured by using a Rabbit Haptoglobin ELISA kit (GenWay). The level of neutrophil gelatinase-associated lipocalin (N-GAL) and vascular endothelial cell growth factor (VEGF) in plasma was measured by using a Rabbit N-GAL ELISA kit and a Rabbit VEGF ELISA kit, respectively (Cusabio). The remaining plasma was stored at -80 °C s. The level of albumin in urine was analyzed at day 1 (G20) and day 10 (G29) using a Rabbit Albumin ELISA kit (Biosite, Nordic Biosite, Sweden). The remaining urine was stored at -80 °C. For all ELISA analysis, all samples were run in duplicates.

### Glomerular sieving coefficient

At end point day 10 (G29), immediately following anesthesia (see above), a single dose of a FITC-Ficoll mixture (FITC-Ficoll 70 and FITC-Ficoll 400 (TdB Consultancy, Uppsala, Sweden), in a 1:24 relationship together with FITC-Inulin (TdB Consultancy, Uppsala, Sweden) was given to the pregnant rabbits by i.v injection in the lateral ear vein. Ficoll is a neutral copolymer of sucrose and epichlorohydrine and is used to measure glomerular permeability to macromolecules by analyzing its steady-state urine-to-plasma concentration ratio as previously described [31, 32]. The single dose contained 0.3 mg of FITC-Ficoll 70, 6.7 mg of FITC-Ficoll 400 and 6.7 mg of FITC-Inulin in a total volume of 1.4 ml. Urine was sampled from the urinary bladder using a syringe at 20 min after the start of the injection. Plasma samples were collected at the same time as the urine samples were collected. A high performance size exclusion chromatography (HPSEC) system (Waters, Milford, MA, USA) was used to determine size and concentration of Ficoll in the urine and plasma samples [33]. The glomerular sieving coefficient (θ) is defined as the filtrate-to-plasma concentration ratio i.e. the concentration of solute in the primary urine over that in plasma. The Ficoll glomerular sieving coefficients (θ) for plasma and urine samples from each rabbit were determined [31, 32].

### Transmission electron microscopy

Biopsies from kidney and placenta (3 x 3 x 3 mm) were fixed for 2 hours at room temperature in fixative (1.5% paraformaldehyde and 1.5% glutaraldehyde in 0.1 M Sörensen buffer pH 7.2), washed and stored overnight at 4 °C in Sörensen buffer. The fixed samples were thereafter prepared for ultrathin sectioning and subjected to transmission electron microscopy as reviewed in [34]. The analysis was carried out manually and blinded by an independent investigator using Adobe Photoshop CS6. For a quantitative evaluation of structural changes of the tissue observed in placenta and kidney, the surface areas of cellular structures such as mitochondria and extracellular matrix space were determined for 60 cellular profiles. Also, the ratio of damaged and intact plasma and nuclear membrane stretches was evaluated.

### Real-time PCR

Total RNA was prepared from frozen biopsies from kidney, liver and placenta using TRIzol (Life Technologies) followed by an E.Z.N.A. Total RNA kit (Omega Bio-Tek, VWR). Reverse transcription with random hexamers was performed using TaqMan Reverse Transcription kit (Applied Biosystems, Roche). Real-time PCR was performed on kidney, liver and placenta for heme oxygenase 1 (HMOX1) with glyceraldehyde 3-phosphate dehydrogenase (GAPDH) as endogenous control using TaqMan Gene Expression Assays specific for rabbit (HMOX1 – Oc03396031_m1, GAPDH – Oc03823402_g1, Life Technologies). The analyses were done by using the relative standard curve method, where a 4-fold dilution series of a cDNA from liver was used as an arbitrary standard to generate an arbitrary unit for each sample as defined by a standard curve for the gene tested. The arbitrary value for each sample was normalized against the value for the endogenous control gene (GAPDH) to give an expression ratio. The ratio was presented as % of the control median. Real-time PCR was performed on liver for rabbit A1M (in-house primers) with rabbit GAPDH (in-house primers) as endogenous control, using iTac Universal SYBR Green PCR Supermix (Bio-Rad). Analyses were done by the ΔΔCt-method [35] with a normalization to GAPDH. For all PCR analysis, all samples were run as duplicates.

### Statistical analysis

All statistical analysis was performed using Origin 8 software (Microcal Northampton, MA, USA). The significance of differences between the groups was evaluated using both Student´s t-test and Mann-Whitney U-test. P-values of p<0.05 were considered significant.

## Results

### Pregnant rabbit model

**Fig 2:**
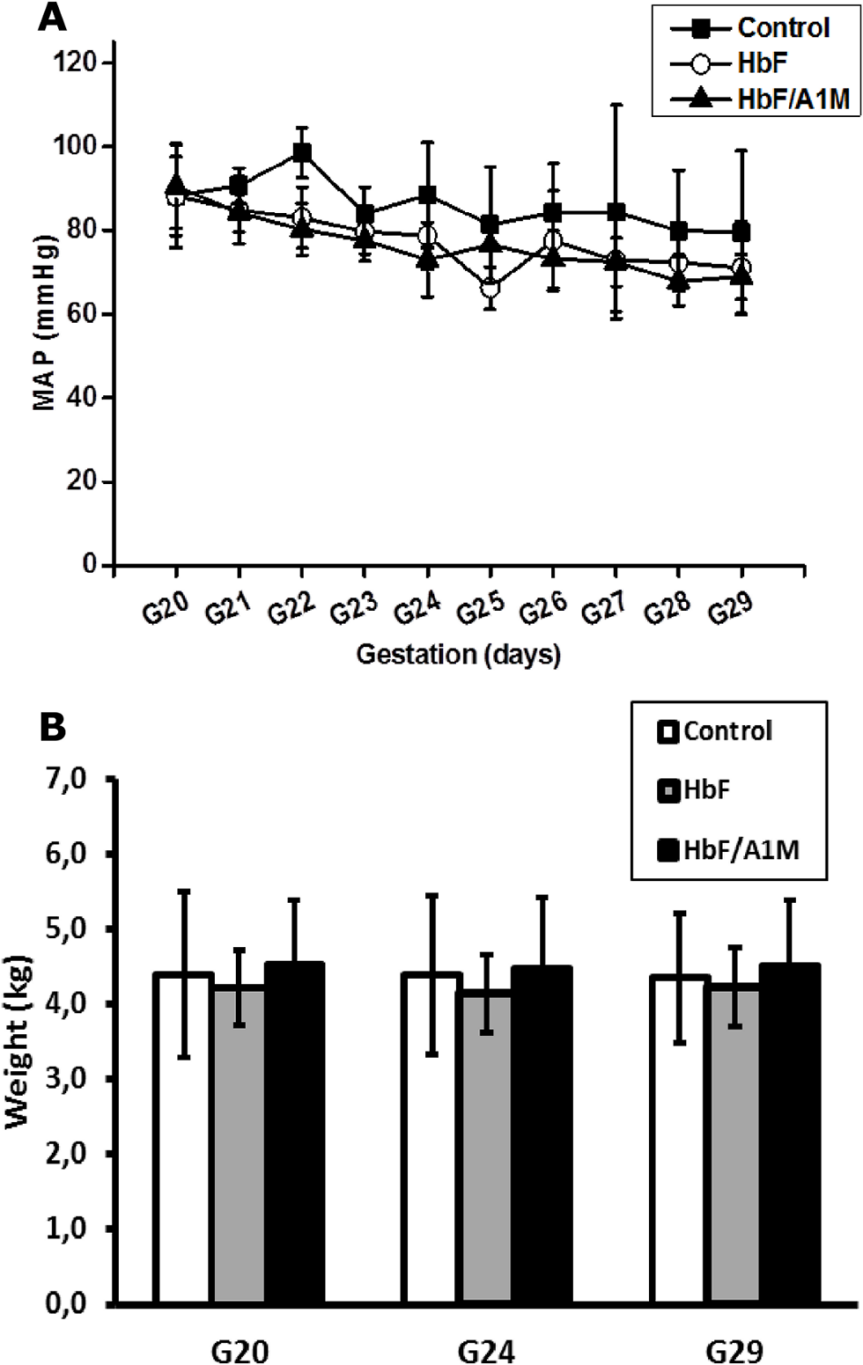
No change in blood pressure after HbF treatment in pregnant rabbits. (A) Shown is the mean arterial pressure measured daily for the different study groups of pregnant rabbits throughout the experiment (G20-G29). Shown is mean ±SD for controls (n = 4), HbF (n = 6) and HbF/A1M (n = 4). (B) No difference in body weight was observed between the different study groups. Shown is mean ±SD for controls (n = 5), HbF (n = 8) and HbF/A1M (n = 6).

Fig 1 shows the experimental setup. The HbF-injections did not result in any changes in mean arterial blood pressure (MAP) (Fig 2A) or in systolic, diastolic, or pulse (data not shown), in the pregnant rabbits throughout gestation. There was no significant difference in body weight between the three study groups during the experimental period (Fig 2B).

### Pregnancy outcome

At the time of termination, there were no significant differences between the groups regarding the number of fetuses and their viability; there was a single dead fetus among five control-litters, one single dead fetus among eight HbF-litters and none among six HbF/A1M-litters. There were no significant differences between the groups in any of the fetal features such as body weight, brain weight, crown-rump length, cranial perimeters or placental weight (Table 1).

**Table 1:** Fetal developmental parameters at time of termination at G29.

Parameter	Control	HbF	HbF/A1M
Number of offspring per dam	9,8 ± 0,8	11,0 ± 2,5	10,0 ± 2,0
Weight (g)	40,7 ± 3,6	42,0 ± 7,6	42,7 ± 6,2
Brain weight (g)	1,2 ± 0,2	1,1 ± 0,2	1,2 ± 0,05
Placental weight (g)	7,7 ± 1,1	7,8 ± 3,1	7,8 ± 2,6
Crown rump lenght (mm)	9,9 ± 0,3	9,9 ± 0,6	9,8 ± 0,4
Anterior posterior cranial perimeter (mm)	30,5 ± 1,4	29,8 ± 1,1	29,6 ± 0,8
Transverse cranial diameter (mm)	18,9 ± 1,0	18,4 ± 0,8	18,0 ± 0,6
Ratio brain weight/weight	0,03 ± 0,006	0,03 ± 0,006	0,03 ± 0,004
Ratio placental weight/weight	0,18 ± 0,03	0,18 ± 0,04	0,18 ± 0,03

Data is presented as mean ± SD. Number of dams: Control (n = 5); HbF (n = 8); HbF/A1M (n = 6).

### Urine analysis

**Fig 3:**
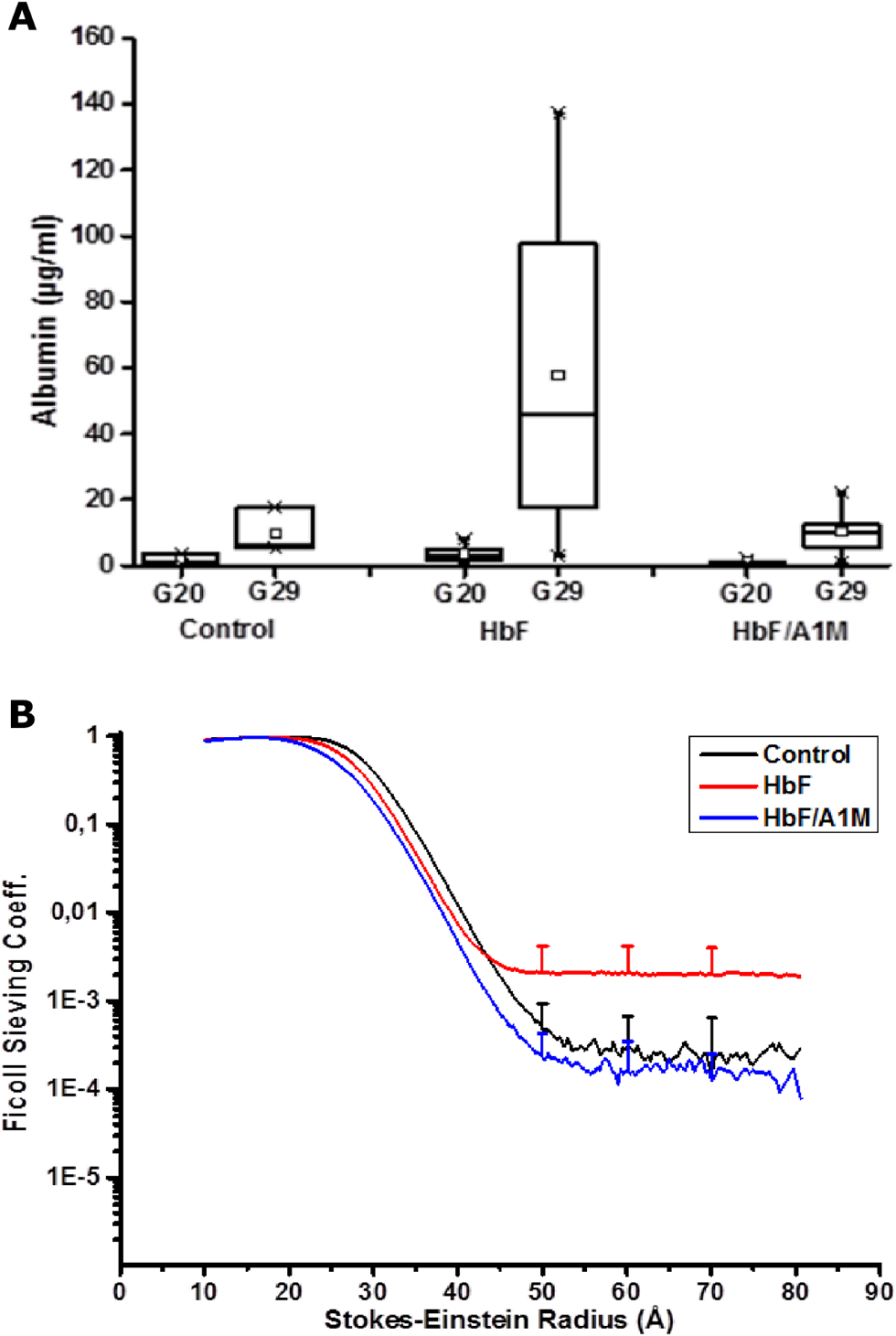
HbF-induced increase in proteinuria and glomerular sieving coefficient is abrogated by A1M. (A) Proteinuria determined by analyzing the levels of the endogenous protein albumin in the urine at G20 and G29. There was a significant increase in albumin levels in the HbF injected group compared to controls (Student´s t-test, p = 0.035). Proteinuria was abrogated by A1M-treatment (Student´s t-test, p = 0.036). Data shown as a boxplot with the 25 and 75 percentile for controls (n = 3), HbF (n = 8) and HbF/A1M (n = 5). (B) Glomerular sieving coefficient *vs*. Stokes-Einstein Radius (Å) for Ficoll molecules of the size 10–80 Å in radius. For HbF-injected animals (red line) there was a significant increase in the sieving coefficient for Ficoll_50-80Å_, compared to control (black line) (Ficoll 70Å: Student´s t-test; p = 0.05). This increase was totally abrogated by A1M-treatment (blue line) (Ficoll 70Å: Student´s t-test; p = 0.03). Data is presented as mean ±SD for controls (n = 3), HbF (n = 8) and HbF/A1M (n = 5).

Urine analysis by the use of a species-specific albumin ELISA indicated a significant leakage of the endogenous protein albumin in the urine of the HbF-treated rabbits compared to controls (Fig 3A). This albumin leakage was inhibited by co-administration of A1M.

### Kidney function

To investigate the effect of HbF on kidney function, the permeability of the glomerular filtration barrier was analyzed with FITC-Ficoll as previously described [31, 32]. Briefly, at time of termination, the sedated animals were given a single dose i.v. injection of a FITC-Ficoll mixture. Urine and blood was collected after 20 minutes and analyzed to determine size and concentration ratio of the Ficoll in urine and plasma. A significant increase in the sieving coefficients for Ficoll_50-80Å_ was seen for the HbF group, compared to controls (Fig 3B), indicating an increase in the number of large pores in the glomerular filter and thereby a defect in the glomerular filtration barrier following injections of HbF. Treatment with A1M completely abrogated the increase in the sieving coefficients seen in HbF animals.

### Structural changes in the kidneys

**Fig 4:**
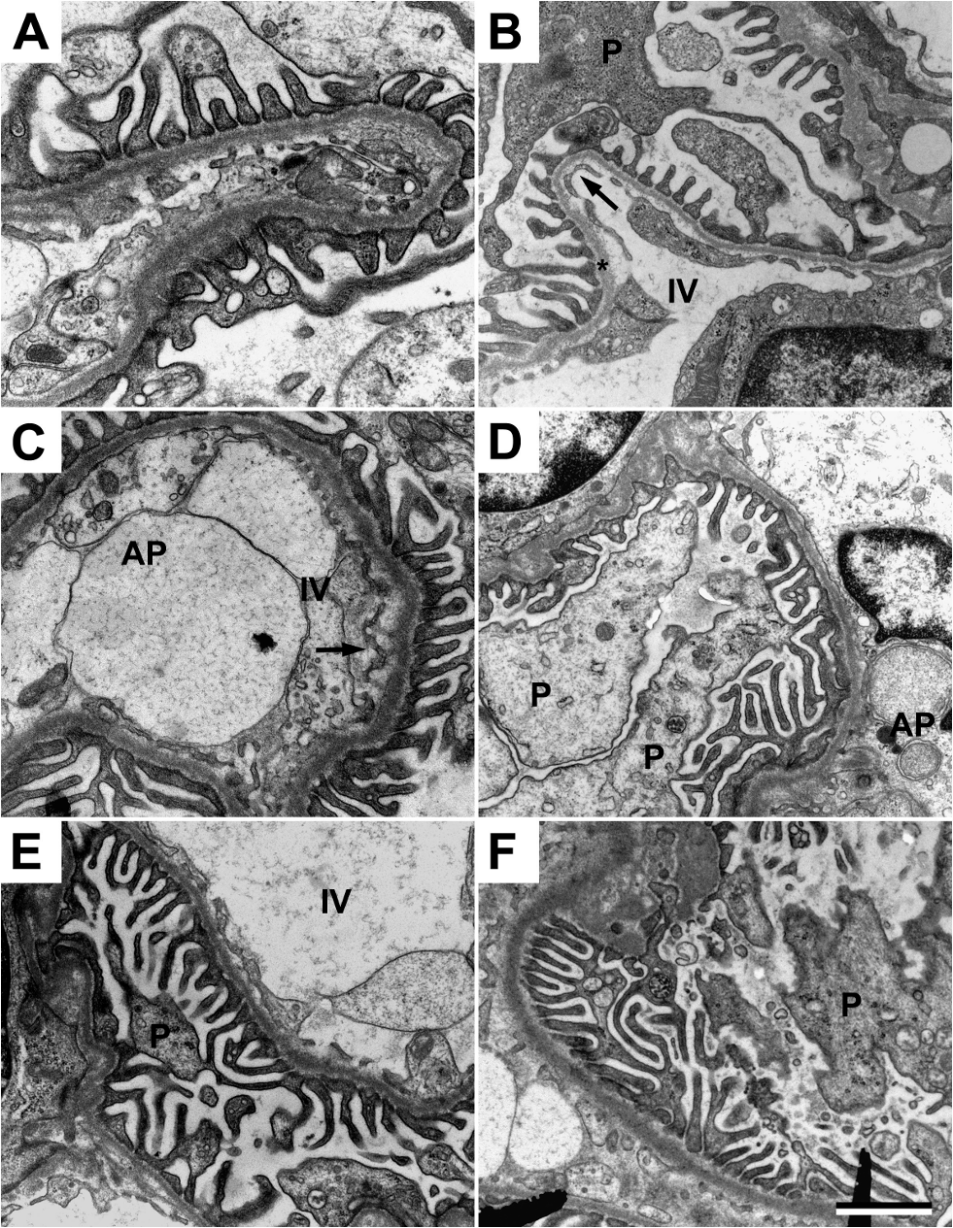
A1M prevents the kidney damages caused by free HbF. Transmission electron microscopy analysis of kidney glomeruli. (A-B) Ultrastructure of the glomerular area in the control animals. Podocytes (P) are without signs of foot process fusions, swelling or villous transformation. Endothelial cells displays a slender morphology and with normal fenestration (arrow). The basal membrane (star) is thin and smooth without structural aberrations. (C-D) Disturbed glomerular morphology in the HbF animals; apoptotic podocytes, abundant membrane bounded saccular structures and apoptotic bodies (AP) in the intravascular space. The glomerular basal membrane is lamellated, resulting in thickening and structural aberrations. The underlying endothelial fenestrations are to a large degree occluded. (E-F) The ultrastructure was significantly normalized after A1M treatment. Note the healthy podocytes, fewer numbers of apoptotic bodies in the intravascular space, thinner basal membranes with smoother texture and normal frequency of fenestration of the endothelial layer. Scale bar 1 μm.

Transmission electron microscopy analysis of kidneys from the control group displayed normal morphology (Fig 4A and 4B and Table 2). Following HbF exposure, there were both intracellular and extracellular changes in the cortex and medulla of the kidneys. The following pathological changes were observed in the glomeruli (Fig 4C and 4D and Table 2); aberrant intravascular membrane systems, high level of intravascular apoptotic vesicles, aberrant degree of endothelial fenestration, resulting in areas of obliterated fenestrations and areas of increased but structurally aberrant fenestrations. The glomerular basal membranes showed structural changes in the form of lamellation of the membranes in a basket weave pattern, resulting in variations in membrane thickness. Signs of podocytes undergoing apoptosis were seen along with multi-vesicular bodies, mitochondrial and endoplasmic reticulum (ER) swelling in the podocytes. The animals treated with A1M displayed a significant reversal of the structural and cellular changes seen following HbF treatment (Fig 4E and 4F and Table 2).

**Table 2:** Quantifications of cellular structures obtained from transmission electron microscopy analysis.

Parameter	Control	HbF	HbF/A1M
**Kidney**			
Extracellular matrix integrity (%)	93 ± 7	22 ± 16^****^	71 ± 11^***^
Glomerular barrier membrane integrity (%)	93 ± 6	21 ± 19^****^	75 ± 7^****^
Nuclear membrane integrity (%)	96 ± 3	45 ± 21^***^	82 ± 5^****^
Mitochondrial cross section (μm)	0.6 ± 0.2	2.0 ± 0.5^****^	0.7 ± 0.3^****^
Intravascular apoptotic bodies (%)	9	82^****^	29^***^
**Placenta**			
Extracellular matrix integrity (%)	90 ± 7	20 ± 18^****^	70 ± 12^****^
Plasma membrane integrity (%)	97 ± 3	23 ± 20^***^	79 ± 8^****^
Nuclear membrane integrity (%)	98 ± 4	40 ± 25^****^	80 ± 5^****^
Mitochondrial cross section (μm)	0.6 ± 0.2	2.1 ± 0.7^****^	0.7 ± 0.4^****^
Extracellular apoptotic bodies (%)	7	88^****^	37^***^

Data is presented as mean ± SD. Statistical analysis were made using Mann-Whitney test for HbF vs control, and HbF/A1M vs HbF.

*** P < 0,001

**** P < 0,0001

### Structural changes in placenta

**Fig 5:**
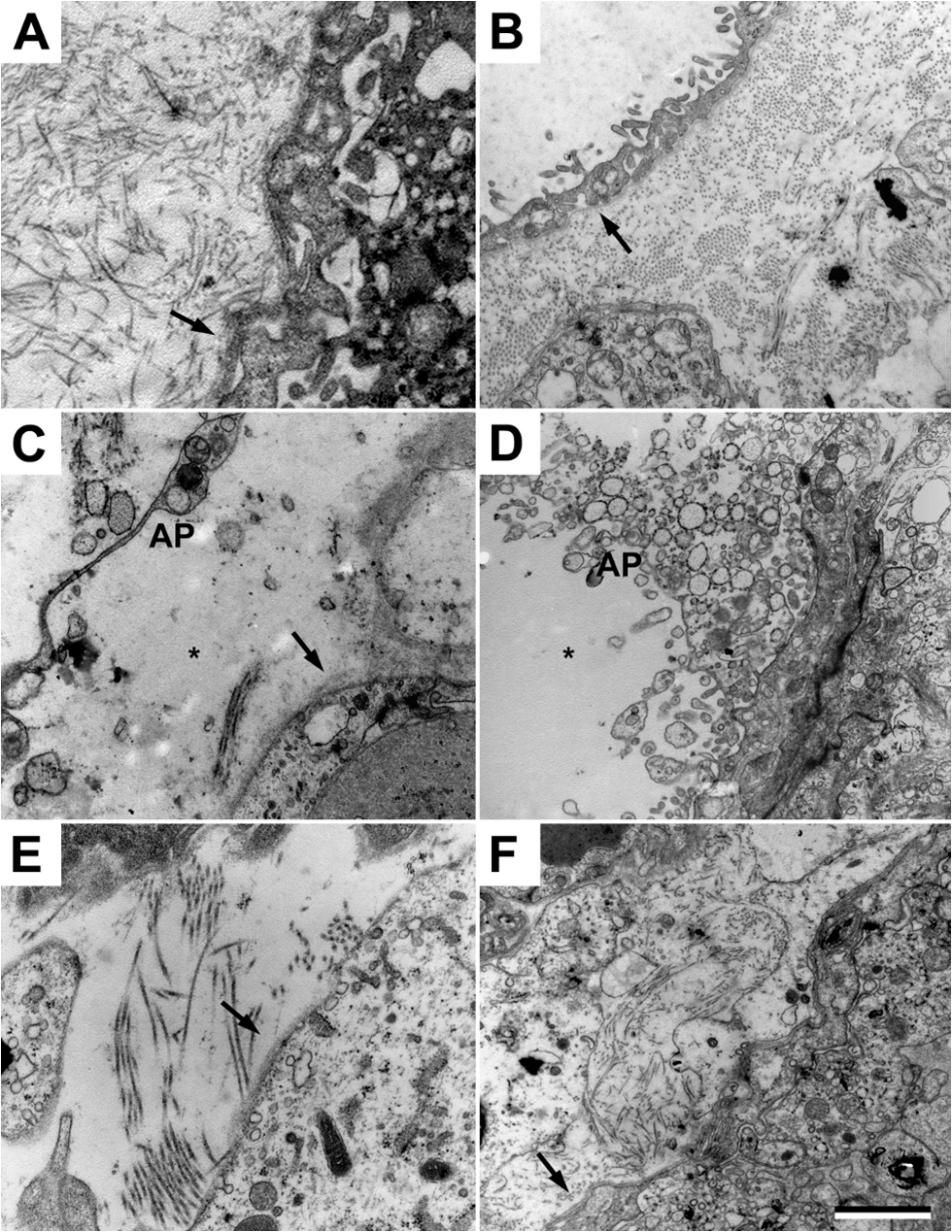
A1M prevents the placental damages caused by free HbF. Transmission electron microscopy analysis of placental tissue. (A-B) Placental tissue from control animals. Normal extracellular matrix with dense bundles of collagen fibers and a normal electron dense barrier (arrow) can be seen. (C-D) Cell-free HbF causes severe damage to the extracellular matrix with significant loss of collagen fibers, increased numbers of extracellular apoptotic bodies (AP), cell debris, disruption of the electron dense barrier and numerous areas of empty extracellular space (star). (E-F) A1M treatment significantly normalized the structural damages. Note the normal bundles of collagen fibers, normal electron dense barrier and reduced numbers of apoptotic bodies in the extracellular space. Scale bar 1 μm.

Transmission electron microscopy analysis of placental tissue from the control group displayed normal morphology (Fig 5A and 5B). HbF exposed animals compared to control showed a loss of the electron dense barrier lining the border towards the extracellular space and loss of the extracellular matrix integrity with an almost complete loss of collagen fibers. This leaves the extracellular space empty except for an increased amount of apoptotic bodies (Fig 5C and 5D). Observed pathological changes in intracellular morphology were aberrant structural integrity of extra-cellular matrix, plasma membranes, nuclear membranes, and mitochondria, as well as an increase in the number of apoptotic bodies (Table 2). The A1M treated animals showed a preserved electron dense barrier, a reduction of apoptotic bodies, and re-organized dense bundles of collagen fibers in the extra-cellular space (Fig 5E and 5F and Table 2).

### Blood analysis

There were no differences in plasma levels regarding whole blood cell counts (S1 Fig) or glucose, ALAT, ASAT, creatinine, urate or LDH (S2 Fig) between the study groups during the experiment. There was a tendency to increased levels of haptoglobin in plasma at the end of the experiment (G29) in the HbF and HbF/A1M groups compared to the control group (S3A Fig). There were no differences in the levels of N-GAL and VEGF in plasma between the study groups at time of termination (S3B and S3C Fig).

### Gene expression

There were no significant differences in HMOX1 gene expression in the liver, kidney and placenta, or in A1M gene expression in the liver between the study groups at the time of termination (S4 Fig). GAPDH was used as an endogenous control and showed no difference in expression between the treatment groups.

## Discussion

Our results show that species-specific cell-free HbF administered to pregnant rabbits during the second half of gestation is associated with tissue damage and disrupted kidney function in a similar way as seen in preeclampsia [2]. Intravenous administration of recombinant human A1M displayed a clear protective effect in both placenta and kidney tissues by reversing the tissue damage caused by administration of cell-free HbF. These protective effects have previously been described in *ex-vivo* human placentas and in a pregnant ewe model for preeclampsia [15, 28], but the present experiment is the first *in vivo* animal model during pregnancy where the A1M mediated alleviation of preeclampsia-like symptoms can be directly linked to an HbF-insult.

In the starvation ewe model, the preeclampsia-like symptoms are suggested to be induced by the low-fed state that involves several physiological changes including hemolysis and an increase in plasma levels of cell-free Hb [27]. The symptoms and organ failures were reversed by A1M. In this study we have specifically assessed the role of cell-free HbF in the pathogenesis of preeclampsia during the second half of gestation and evaluated the therapeutic potential of A1M to relieve induced symptoms. We attempted to establish a rabbit model mimicking the human symptoms at stage two of preeclampsia by administering species-specific cell-free HbF starting mid-gestation until term. The rabbit is used in several well-established related models to study placental circulation, placental transfer and fetal aspects such as IUGR and brain injury [36–38]. The rabbit placenta has similar features to the human placenta; it is discoid, villous and hemochorial [39]. The current rabbit model developed typical symptoms seen in preeclampsia, such as tissue damage to the placenta and functional and structural damages to the kidneys shown as proteinuria and disruption of the glomerular filtration barrier. However, the rabbits did not display elevated blood pressure, a hallmark for preeclampsia. Previous studies attempting to elevate blood pressure in pregnant dams by administering vasoactive hormones have had little or no success [40], and it has been shown that pregnant rabbits have lower blood pressure compared to non-pregnant rabbits. It was speculated that there is a pregnancy-related protective mechanism, since the sensitivity for the vasoactive hormones increased after parturition. This could be a contributing factor to the lack of blood pressure elevation in this model after administration of cell-free HbF.

The therapeutic effects of A1M support the role of this protein as a protective heme- and radical-binding protein. A1M, although mainly synthesized by the liver [41], and secreted to the blood, has an important role in the extravascular compartments where it functions as a cell and tissue housekeeping protein with a central role in clearing extravascular fluids of heme and oxidative waste products, and repairing lesions caused by oxidative stress [26]. A1M has been shown *in vitro* to have protective roles by preventing intracellular oxidation, inhibit cell lysis, clearing cells from bound heme and it also inhibits mitochondria swelling [23, 42, 43]. A1M also protects against bystander cell killing *in vitro*, and through tissue housekeeping clears free radicals and oxidants that are released from apoptotic cells [44, 45]. As a cell and tissue protective free radical- and heme scavenger, A1M has the potential to be used as a therapeutic drug for treatment or prophylaxis of pathological conditions associated with oxidative stress. The therapeutic potential could be seen in this work as a protection of pregnant rabbits infused with cell-free HbF, both in the placenta and the kidneys. In the placenta, the extracellular matrix was protected by A1M and the destruction of collagen fibrils inhibited. It has previously been shown that A1M can induce the repair of collagen fibers even after the destruction has taken place [42]. In the kidneys, A1M restored the HbF-induced increase in glomerular permeability to normal levels and significantly restored the structural damages caused by cell-free HbF.

Some obvious limitations of the study need further discussion. I) No hypertension was seen after the HbF-infusion. The increase in maternal blood pressure in preeclampsia occurs downstream of events taking place during the first stage of the disease. As we exposed the maternal circulation to cell-free HbF starting at mid-gestation we are only mimicking stage two of the disease. Hence, the timing of the HbF exposure might not have been optimal. Thereby, mid-gestation HbF exposure in the maternal circulation may fall short in provoking a blood pressure elevation. II) The HbF was infused directly into the maternal circulation rather than leaking from the placenta as suggested to occur in preeclampsia [14, 15, 18]. The overproduction of HbF described in the preeclamptic placenta may cause local oxidative stress, release of angiogenic factors and breach of the placental barrier [16–18]. By infusing the HbF into the maternal circulation we achieve structural tissue damage to the placenta, but other reported manifestations of the placental insult may not have been induced.

In conclusion, this model demonstrates that free HbF administered to the pregnant rabbit leads to structural and functional damage to the rabbit kidney and structural changes in the placenta which resembles those found in human patients with preeclampsia. Hence, this model may be used to study stage two of preeclampsia in general, and kidney and placental function in particular. The induced tissue damage and organ disruption was relieved by co-administration of A1M. This study provides preclinical evidence supporting further examination of A1M as a potential new therapy for preeclampsia.

## Supporting Information

S1 Fig No differences between the study groups when analyzing whole blood cell counts. Levels of (A) white blood cells, (B) lymphocytes, (C) monocytes, (D) neutrophils, (E) red blood cells, (F) platelets, (G) hemoglobin, (H) hematocrit, (I) mean cell volume and (J) mean cell hemoglobin. Data is presented as mean ± SD. Controls (n = 5), HbF (n = 8) and HbF/A1M (n = 6).
(TIF)

S2 Fig No differences between the study groups when analyzing levels of (A) ALAT, (B) ASAT, (C) LDH, (D) creatinine, (E) urate and (F) glucose. Data is presented as mean ± SD. Controls (n = 5), HbF (n = 8) and HbF/A1M (n = 6).
(TIF)

S3 Fig (A) A tendency to increased plasma haptoglobin levels at G29 in the HbF and HbF/A1M group compared to control. Data is shown as box plots, with the 25 and 75 percentile. (B) No difference in N-GAL plasma levels between the groups during the experiment. Data is shown as mean ±SD. (C) No difference in VEGF plasma levels between the groups during the experiment. Data is shown as mean ±SD. For A-C: Control (n = 5), HbF (n = 8) and HbF/A1M (n = 6).
(TIF)

S4 Fig No significant difference in HMOX1 gene expression between groups in (A) kidney, (B) placenta and (C) liver. (D) No significant difference between groups in A1M gene expression in liver. Control (n = 5), HbF (n = 8) and HbF/A1M (n = 6).
(TIF)
