# Supplementary figures and images for: A1M Ameliorates Preeclampsia-Like Symptoms in Placenta and Kidney Induced by Cell-Free Fetal Hemoglobin in Rabbit
Source: PLoS One. 2015 May 8;10(5):e0125499. doi: 10.1371/journal.pone.0125499 (PMC4425457; doi:10.1371/journal.pone.0125499)

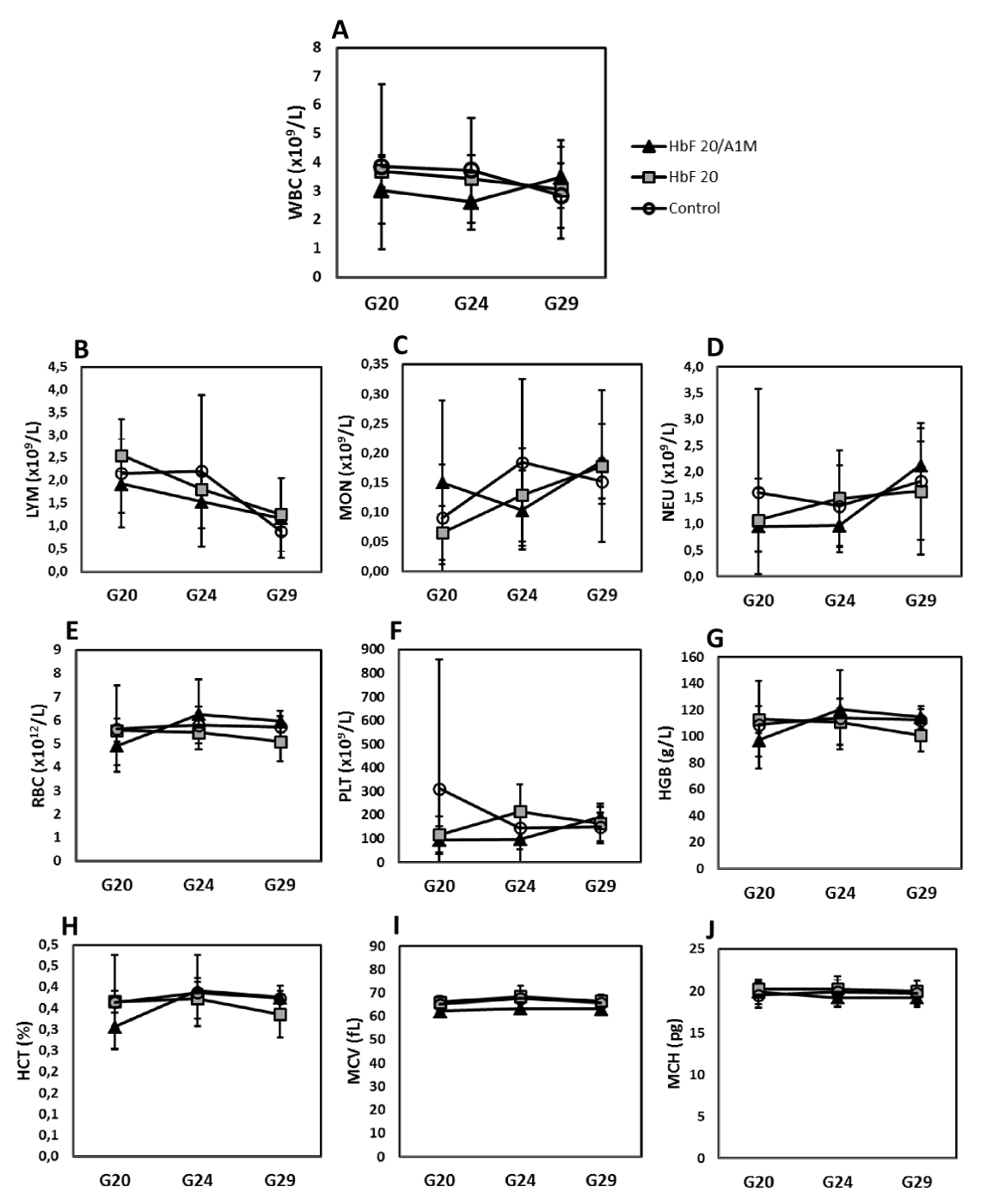

Supplement: S1 Fig — Levels of (A) white blood cells, (B) lymphocytes, (C) monocytes, (D) neutrophils, (E) red blood cells, (F) platelets, (G) hemoglobin, (H) hematocrit, (I) mean cell volume and (J) mean cell hemoglobin. Data is presented as mean ± SD. Controls (n = 5), HbF (n = 8) and HbF/A1M (n = 6). (TIF) [file pone.0125499.s001.tif]

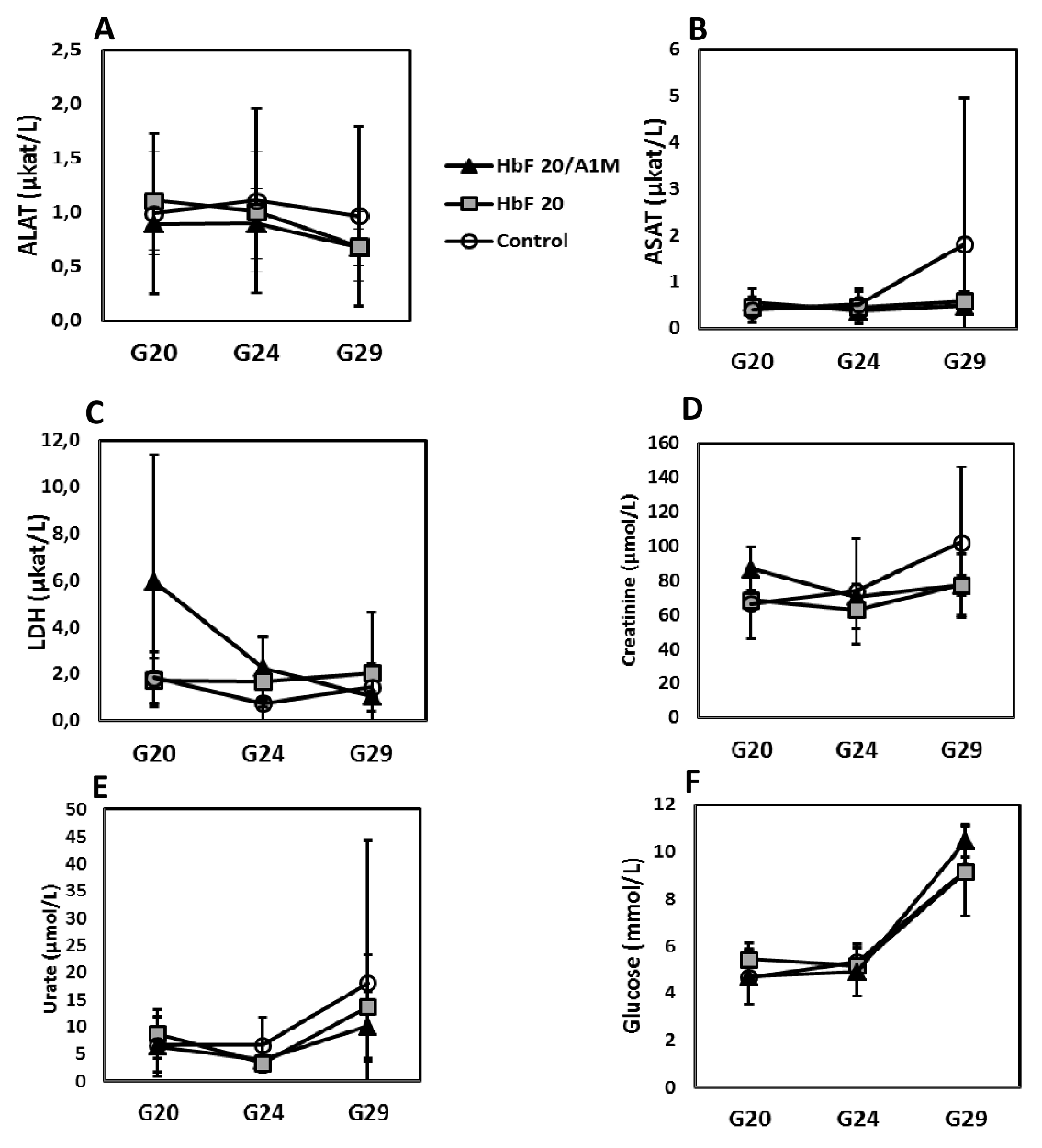

Supplement: S2 Fig — Data is presented as mean ± SD. Controls (n = 5), HbF (n = 8) and HbF/A1M (n = 6). (TIF) [file pone.0125499.s002.tif]

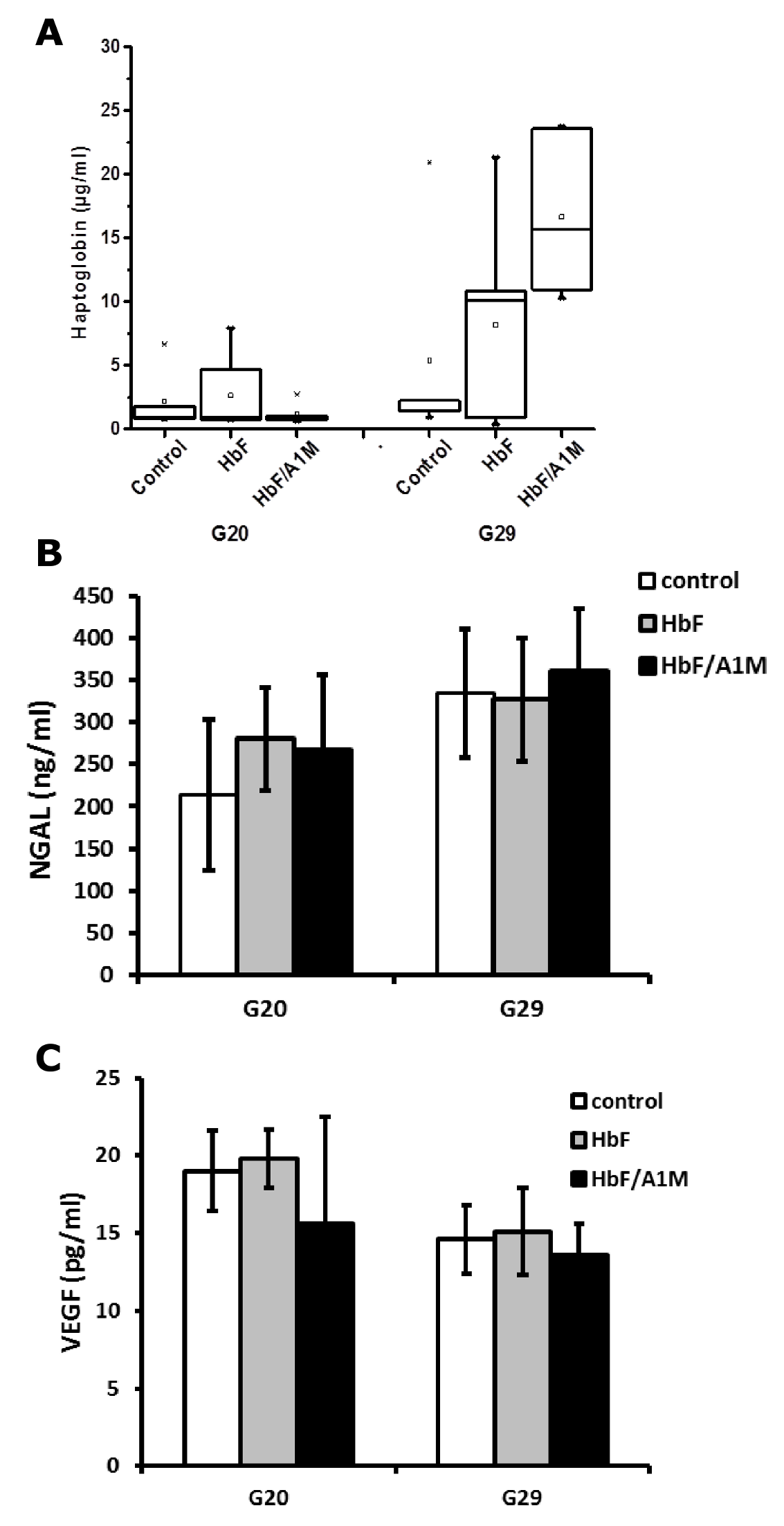

Supplement: S3 Fig — Data is shown as box plots, with the 25 and 75 percentile. (B) No difference in N-GAL plasma levels between the groups during the experiment. Data is shown as mean ±SD. (C) No difference in VEGF plasma levels between the groups during the experiment. Data is shown as mean ±SD. For A-C: Control (n = 5), HbF (n = 8) and HbF/A1M (n = 6). (TIF) [file pone.0125499.s003.tif]

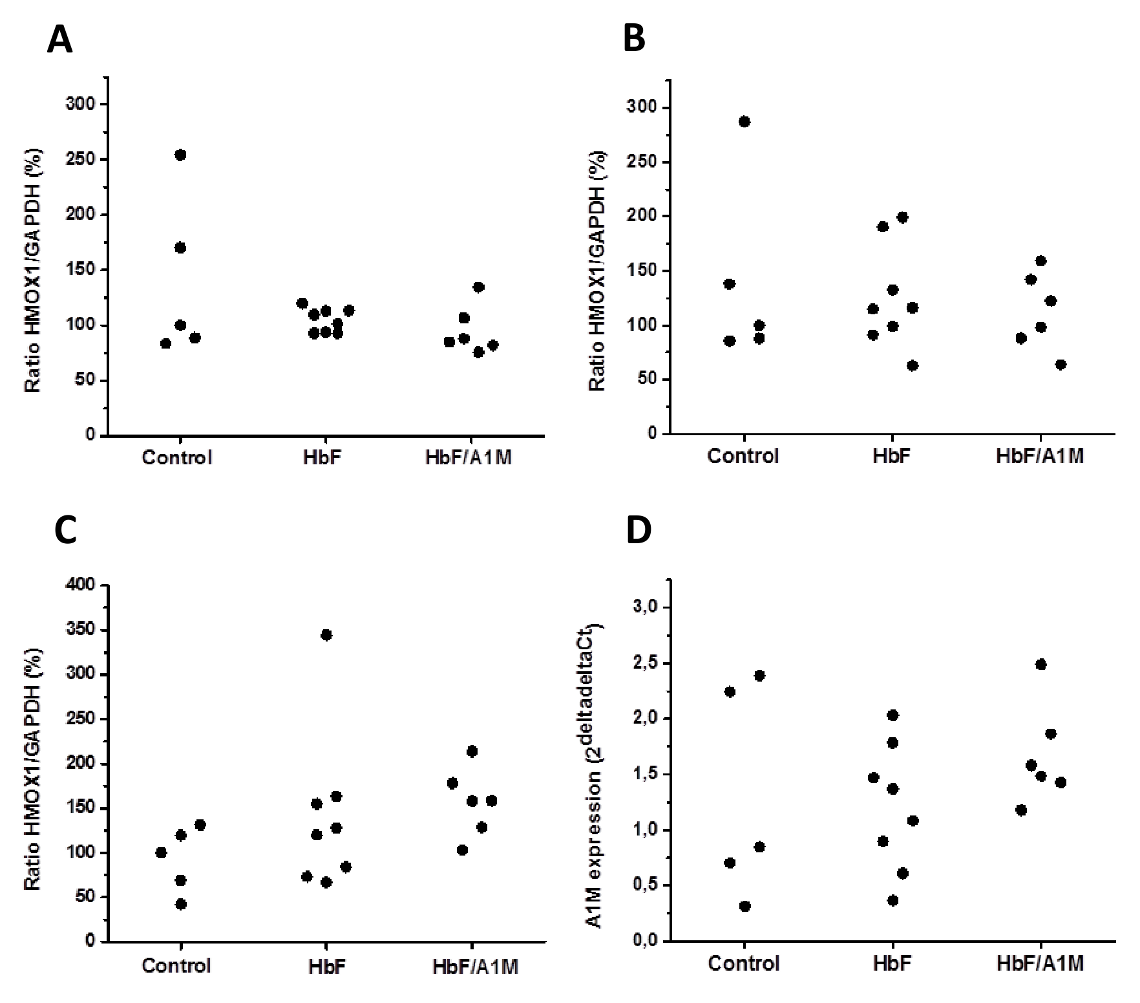

Supplement: S4 Fig — (D) No significant difference between groups in A1M gene expression in liver. Control (n = 5), HbF (n = 8) and HbF/A1M (n = 6). (TIF) [file pone.0125499.s004.tif]
